# Acceptance and Commitment Therapy Delivered via a Mobile Phone Messaging Robot to Decrease Postoperative Opioid Use in Patients With Orthopedic Trauma: Randomized Controlled Trial

**DOI:** 10.2196/17750

**Published:** 2020-07-29

**Authors:** Chris A Anthony, Edward Octavio Rojas, Valerie Keffala, Natalie Ann Glass, Apurva S Shah, Benjamin J Miller, Matthew Hogue, Michael C Willey, Matthew Karam, John Lawrence Marsh

**Affiliations:** 1 Department of Orthopaedics University of Pennsylvania Philadelphia, PA United States; 2 Department of Orthopedic Surgery Washington University School of Medicine St Louis, MO United States; 3 Department of Orthopaedics and Rehabilitation University of Iowa Hospitals and Clinics Iowa City, IA United States; 4 Children’s Hospital of Philadelphia Main Campus Division of Orthopaedics Philadelphia, PA United States

**Keywords:** acceptance and commitment therapy, opioid crisis, patient-reported outcome measures, postoperative pain, orthopedics, text messaging, chatbot, conversational agents, mHealth

## Abstract

**Background:**

Acceptance and commitment therapy (ACT) is a pragmatic approach to help individuals decrease avoidable pain.

**Objective:**

This study aims to evaluate the effects of ACT delivered via an automated mobile messaging robot on postoperative opioid use and patient-reported outcomes (PROs) in patients with orthopedic trauma who underwent operative intervention for their injuries.

**Methods:**

Adult patients presenting to a level 1 trauma center who underwent operative fixation of a traumatic upper or lower extremity fracture and who used mobile phone text messaging were eligible for the study. Patients were randomized in a 1:1 ratio to either the intervention group, who received twice-daily mobile phone messages communicating an ACT-based intervention for the first 2 weeks after surgery, or the control group, who received no messages. Baseline PROs were completed. Two weeks after the operative intervention, follow-up was performed in the form of an opioid medication pill count and postoperative administration of PROs. The mean number of opioid tablets used by patients was calculated and compared between groups. The mean PRO scores were also compared between the groups.

**Results:**

A total of 82 subjects were enrolled in the study. Of the 82 participants, 76 (38 ACT and 38 controls) completed the study. No differences between groups in demographic factors were identified. The intervention group used an average of 26.1 (SD 21.4) opioid tablets, whereas the control group used 41.1 (SD 22.0) tablets, resulting in 36.5% ([41.1-26.1]/41.1) less tablets used by subjects receiving the mobile phone–based ACT intervention (*P*=.004). The intervention group subjects reported a lower postoperative Patient-Reported Outcome Measure Information System Pain Intensity score (mean 45.9, SD 7.2) than control group subjects (mean 49.7, SD 8.8; *P*=.04).

**Conclusions:**

In this study, the delivery of an ACT-based intervention via an automated mobile messaging robot in the acute postoperative period decreased opioid use in selected patients with orthopedic trauma. Participants receiving the ACT-based intervention also reported lower pain intensity after 2 weeks, although this may not represent a clinically important difference.

**Trial Registration:**

ClinicalTrials.gov NCT03991546; https://clinicaltrials.gov/ct2/show/NCT03991546

## Introduction

### Opioid Medication Issues

Public health concerns regarding opioid medications persist, and health care systems are currently seeking solutions to the ongoing epidemic [1]. In 2017, the rate of drug overdose deaths involving opioids in the United States increased by 12%, totaling 47,600 cases, and prescription opioid medications accounted for over 17,000 of these [2]. Even small amounts of additional daily opioid utilization (10 morphine milliequivalents [MME]) by patients can lead to an increased risk of long-term misuse [3]. In addition, every week of continued opioid utilization represents an increased risk of eventual misuse by patients [3]. Previous studies have found that orthopedic trauma patients use a decreasing amount of opioid medication in the first 2 postoperative weeks, with 6-15 days being the optimal opioid use period [4,5]. In line with these findings, previous studies have used a 2-week postoperative period to assess opioid medication consumption in surgical patients [4].

### Patient-Reported Outcomes

Patient-reported outcomes (PROs) allow patients to quantify aspects of their orthopedic condition in a standardized fashion [6,7]. These are important tools for determining the efficacy of health care treatments and assessment of clinical research and can be used in determining compensation for health care services provided [6-8]. The National Institutes of Health developed the Patient-Reported Outcome Measure Information System (PROMIS) tools to advance PROs by creating question banks that could be used for many major health issues [9]. The PROMIS Pain Intensity 1A Short form, PROMIS Pain Intensity 3A Short form, PROMIS Pain Interference 8A Short form, and PROMIS Emotional Distress-Anxiety 8A Short form all employ a fixed low number of questions that are highly reliable when compared with their respective domain’s full item bank, making them excellent tools for both patients and clinicians [9,10].

### Acceptance and Commitment Therapy

Acceptance and commitment therapy (ACT) is a cognitive contextual behavioral therapy that employs a pragmatic approach to help individuals decrease pain and live according to self-identified personal values [11,12]. The goal of ACT is to augment an individual’s psychological flexibility, thus improving their life according to 6 core cognitive processes: acceptance, defusion, contact with the present moment, self-as-context, values, and committed action (Table 1) [11]. Employing these cognitive processes to increase psychological flexibility allows people to choose their actions based on what they value most, resulting in decreased avoidance behaviors and negative cognitive associations [11]. ACT has proven to be effective across multiple studies and patient populations in the treatment of pain [13]. Several studies report a high value for ACT in the management of chronic pain when compared with standard pharmacological treatment alone [13-16]. Moreover, earlier cessation of pain and opioid utilization in at-risk orthopedic surgery patients receiving office-based ACT interventions has also been reported [17]. However, traditional ACT interventions require a clinic-based, interdisciplinary team approach, which is not always feasible for both patients and health care systems [17,18].

**Table 1:** Acceptance and commitment therapy core principles with associated messages.

Core principle	Example mobile phone message
Values: know what matters most	Stop for a moment and remember the 3 values you identified earlier today. Remind yourself how important these values are in your life. As your day comes to an end, remember that YOU are in control of the thoughts that exist in your mind. We encourage you to spend time thinking about your 3 core values identified earlier today.
Acceptance: setting expectation that pain is a part of surgery	Feelings of pain and feelings about your experience of pain are normal after surgery. Acknowledge and accept these feelings as part of the recovery process. Remember how you feel now is temporary and your healing process will continue. Call to mind pleasant feelings or thoughts that you experienced today.
Present moment awareness: mindfulness and awareness for our thoughts in the present moment	Awareness of the present moment and your breathing may change with pain-related emotions or thoughts. Remember you can always count on your breathing to bring you back to the present moment and help you move through your current experience of pain.
Self-as-context: awareness of what is being observed and noticed by ourselves	We cannot change that a feeling or thought may arise, but we can choose how we respond to our feelings and thoughts. Remember that dwelling on pain, discouraging feelings, and thoughts after surgery are NOT consistent with your life goals and values. Observe things that try to move you away from your values and only act on things that are compatible with who you want to be and what matters to you.
Committed action: doing what it takes to live according to our values	Healing after surgery requires you to act. We previously discussed your life goals, meaning, and purpose. Take action today and move closer toward what you want in life. Recognize that pain may be present but make the choice that it will not impede your progress toward what you really want in life. Be present in the moment and ensure your actions remain true to what you want most. All actions you make no matter how small, are an important steppingstone on your road to recovery.
Defusion: watch your thinking and interact with thoughts in a way consistent with your values	If you ever feel pain after surgery know that the feeling is real but what it actually represents is not what you might think. Our mind is capable of making us feel pain, even though there is no damage going on in our body. Pause, become more aware in the moment and chose a skillful response that will help you move toward your overall goals and values.

### Mobile Phone Messaging Communication

Evolving communication methods, such as automated mobile phone messaging [4,19-21], for health care purposes are increasingly important, as patients prefer these communication methods for delivering and receiving medical information [22]. Software-driven, automated mobile phone messaging robots (also called chatbots or conversational agents) are low-cost tools that can deliver predefined text-based information and receive incoming responses with high reliability when patients either prefer it or it is necessary to communicate at distance [19,23]. This technology demonstrates high efficacy as part of the treatment of conditions ranging from hypertension to substance abuse [24-26], and it has also proved effective in increasing perioperative communication after hip and knee arthroplasty [27] and collecting pain and opioid medication data from patients following orthopedic trauma and hand procedures [4,19]. In addition, automated mobile phone messaging robots have been validated in the collection of PROs in patients undergoing orthopedic hand [20] and hip preservation procedures [23]. Although mobile phone messaging robots (*Chat bots*) provide the benefit of communicating with patients at distance with no need for human intervention, they do introduce delivery of health care that lacks human interaction with unknown effects [28].

Health care teams caring for patients with traumatic orthopedic injuries have traditionally used opioid medication in the postoperative setting, and these patients are at risk for prolonged opioid utilization in the postoperative period. We theorized that the combination of ACT delivered via automated mobile phone messaging may help to decrease pain and opioid utilization in the acute postoperative setting. The aim of this prospective randomized controlled trial was to evaluate the effectiveness of ACT delivered via an automated mobile messaging robot on (1) decreasing early postoperative opioid utilization and (2) pain-related PROs in the first 2 weeks following surgery for acute traumatic orthopedic injuries.

## Methods

### Study Approval

This randomized controlled trial was registered with ClinicalTrials.gov (NCT03991546) and reporting is consistent with the Consolidated Standards of Reporting Trials guidelines (Multimedia Appendix 1) [29]. The study was performed at a single center university hospital in Iowa City, Iowa, United States. Ethical approval of this study was provided by the University of Iowa institutional review board, and the study was determined to be Health Insurance Portability and Accountability Act compliant.

### Recruitment and Randomization

Adults presenting to a university hospital level 1 trauma center indicated for operative fixation of a traumatic upper or lower fracture were considered for the study (Table 2). The exclusion criteria are listed in Textbox 1. Eligible patients were approached before surgery by a research assistant in a private room. Individuals not excluded by screening questions and interested in participating underwent the informed consent process (Textbox 1). During consent, all subjects were informed of the outcomes of interests, different study arms, and that no changes would be made to their care in terms of postoperative medication, regardless of study participation.

Participants were randomized to either the control or intervention group using a standard web-based random number generator with a range set from 1 to 10 and a 1:1 ratio by a research assistant. Owing to the nature of this study, subjects and the enrolling research assistant were not blinded to the participant’s study group following randomization.

At the time of consent, subjects were required to complete paper forms comprising a basic demographics questionnaire and baseline PROs consisting of the PROMIS Pain Intensity 1A Short form, PROMIS Pain Intensity 3A Short form, PROMIS Pain Interference 8A Short form, and PROMIS Emotional Distress-Anxiety 8A Short form (Multimedia Appendix 2). Following completion of all PROs, participants were randomized to their study group. Subjects who received an odd number from the 1 to 10 range set on the random number generator were placed in the intervention group, whereas subjects given an even number were placed in the control group.

**Table 2:** Injury by final study group (N=76).

Injury	Acceptance and commitment therapy group participants, n	Control group participants, n
Acetabular fracture	1	1
Ankle fracture	15	14
Calcaneus fracture	0	1
Clavicle fracture	0	1
Distal femur fracture	0	2
Distal humerus fracture	1	0
Elbow fracture	2	5
Femoral neck fracture	2	2
Femoral shaft stress fracture	0	1
Intertrochanteric hip fracture	1	0
Navicular fracture	1	0
Patella fracture	1	0
Polytrauma^a^	2	1
Proximal humerus fracture	2	1
Subtrochanteric femur fracture	0	2
Tibial plateau fracture	4	3
Tibial plafond fracture	6	4

^a^Polytrauma was defined as a patient with a fracture to more than one upper or lower extremity.

**Exclusion criteria.**

Screening questions

- No personal mobile phone with text messaging capabilities
- Poor familiarity reading or sending mobile messages

Patient factors

- Open fracture
- Infection at the fracture site
- Prior fracture temporization with an external fixator
- Revision surgery for nonunion or hardware failure
- Bilateral upper extremity injuries impeding their ability to use a mobile phone
- Fractures of the distal hand or distal foot only
- Admission to an intensive care unit
- Current cancer diagnosis or dementia
- Inpatient for more than 7 days of the 2-week study period
- Discharged without an opioid pain medication prescription
- Initial plan for operative fixation changed to treatment with joint arthroplasty

### Study Interventions

The intervention group received twice-daily, text-based mobile messages communicating an ACT-based intervention for the first 2 weeks following surgery (Multimedia Appendix 3). Control group subjects did not receive the ACT intervention or any other form of mobile message communication. The mobile messaging ACT protocol consisted of twice per day mobile messages, morning and evening, starting on postoperative day (POD) 1 and ending on POD 14. These mobile phone messages provided participants with an ACT-based intervention that was developed in collaboration with a pain psychologist (VK) specializing in ACT for chronic pain. These messages used all the principles presented in Table 1 with the objective of helping recipients understand and develop better coping skills in relation to their postoperative pain. An example message from day 1 is as follows:

Maintaining focus on what you value most in life is sometimes difficult after surgery. Do not let the momentary discomforts due to surgery take away from what you want most in life. Pick 3 things that matter most to you in life. Remind yourself of these 3 things you value most during your recovery process.

Outside of the mobile messaging intervention, both groups received the same standard postoperative care, health care team communications, and instructions for completing the study follow-up.

A chart review was performed to collect demographic information such as subject age, comorbid conditions, and preoperative outpatient opioid medication prescriptions for treatment of their current traumatic orthopedic injury. All subjects, regardless of group, were seen by a research team member after surgery to review which of their discharge medications was the medication of interest for the study and to confirm that the intervention group subjects received their first mobile phone ACT message. Participants in both groups were instructed to have their opioid medication bottle available at follow-up to confirm their opioid tablet consumption. Owing to the changes in health care teams, staff preferences, and allergies, the opioid pain medications administered at discharge were not standardized between study groups. Following discharge on POD 14, subjects were contacted by phone or seen in the clinic by the research team for follow-up. At this time, the subjects’ opioid pain medication consumption was assessed, and they completed a second set of PROs.

### Outcome Measures

The primary outcome of this study was the amount of opioid pain medication consumed by subjects, and the secondary outcomes analyzed were net changes from baseline PRO scores at the 2-week follow-up.

The method that participants employed to report their opioid medication consumption and how PROs were captured during follow-up were recorded (Table 3). Subjects using their pill bottle to confirm the remaining number of opioid pain medication tablets from their discharge prescription on POD 14 were denoted as reporting a pill count. Cases where subjects or their care facility kept a log of tablet consumption were classified as reporting a daily log. Subjects reporting the number of tablets they used without the use of a log or pill count were designated as providing an estimate. The percentage of opioid pain medication used, total MME, and percentage of available MME consumed were calculated. The mean number of opioid pain medication tablets and MME used by the subjects were compared between groups. The raw scores for PROs were converted to corresponding *t*-scores using the appropriate PROMIS scoring manual [30]. The changes in PROs from baseline to POD 14 were also calculated by subtracting POD 14 scores from baseline scores, as higher *t*-scores signify a poorer outcome; thus, lower scores on POD 14 indicate an improvement from baseline PROs. The mean PRO scores and changes were compared between the groups.

**Table 3:** Comparison of subject demographics by enrolled study group.

Subject characteristic			Acceptance and commitment therapy group (n=42)		Control group (n=40)		*P* value
Age (years), mean (SD)			45.5 (15.9)		48.7 (14.6)		.41
BMI (kg/m^2^), mean (SD)			30.5 (7.3)		31.1 (8.3)		.94
**Sex, n (%)**						.65	
	Female	22 (52)		19 (48)			
	Male	20 (48)		21 (52)			
Subjects removed or lost to follow-up, n (%)			4 (10)		2 (5)		N/A^a^
Preoperative PROMIS^b^ Pain Intensity 1A Score, mean (SD)			5.4 (2.9)		6.2 (2.6)		.20
Preoperative PROMIS Pain Intensity 3A Score, mean (SD)			54.9 (7.3)		57.1 (8.2)		.23
Mean Preoperative PROMIS Pain Interference 8A Score, mean (SD)			63.6 (11.4)		66.1 (8.4)		.30
Mean Preoperative PROMIS Emotional Distress-Anxiety 8A Score, mean (SD)			56.5 (11.4)		56.5 (9.2)		.99
Days between injury and surgery, mean (range)			4 (1-33)		3 (1-50)		.26
**Disposition^c^, n (%)**						.68	
	Home	36 (95)		34 (90)			
	Skilled nursing facility or acute rehabilitation	2 (5)		4 (10)			
**Ethnicity/race, n (%)**						.86	
	White	37 (88)		35 (88)			
	African American	4 (10)		4 (10)			
	Asian	1 (2)		0 (0)			
	Hispanic	0 (0)		1 (2)			
Preoperative outpatient opioid prescription, n (%)			23 (55)		17 (43)		.17
Current psychiatric diagnosis, n (%)			15 (36)		9 (23)		.14
History/current substance abuse diagnosis, n (%)			8 (19)		3 (8)		.10
Diabetes diagnosis, n (%)			2 (5)		7 (18)		.15
Current smoker, n (%)			7 (17)		9 (23)		.57
Current lumbago diagnosis, n (%)			1 (2)		2 (5)		>.99
History of/current chronic pain diagnosis, n (%)			10 (24)		8 (20)		.59
Number of opioid tablets prescribed^c^, mean (SD)			58.8 (27.3)		61.6 (22.0)		.62
**Opioid utilization reporting method^c^, n (%)**						.47	
	Pill count	34 (90)		30 (79)			
	Daily log	3 (8)		6 (16)			
	Estimate	1 (2)		2 (5)			
Patients filling only one postoperative opioid prescription^c^, n (%)			34 (90)		34 (90)		>.99

^a^N/A: not applicable.

^b^PROMIS: Patient-Reported Outcome Measures Information System.

^c^Data calculated using final study population only (n=38).

### Statistical Analysis

Participant characteristics were described using mean (SD) or median (minimum to maximum) for continuous variables and frequencies (percentages) for categorical variables. Visual review of histograms and the results of the Shapiro-Wilk test of continuous variables revealed that only age and BMI were not normally distributed. Between-group differences were evaluated using *t* tests or Wilcoxon rank-sum tests (age and BMI) for continuous variables and chi-square or exact tests for categorical variables, as appropriate.

To evaluate whether the intervention versus control group had a lower opioid use on average, we determined the number of tablets and MME taken in each group and compared means using *t* tests. Using a previous study of opioid medication usage in orthopedic trauma patients [4], the sample size estimated to observe a 30% decrease in opioid utilization among 2 groups required a total of 74 subjects to achieve 80% power at an alpha of .05. The percent decrease is calculated using the formula 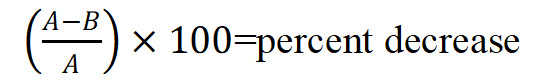. A separate power analysis was calculated for the PRO portion of the study, and it was determined that a total of 36 subjects would provide 80% power to detect a 10-point difference (1 standard deviation) in *t*-scores for the PROMIS instruments at an alpha level of .05. Statistical analyses were performed using SAS software version 9.4 (SAS Institute, Inc).

## Results

### Study Participants

**Figure 1:**
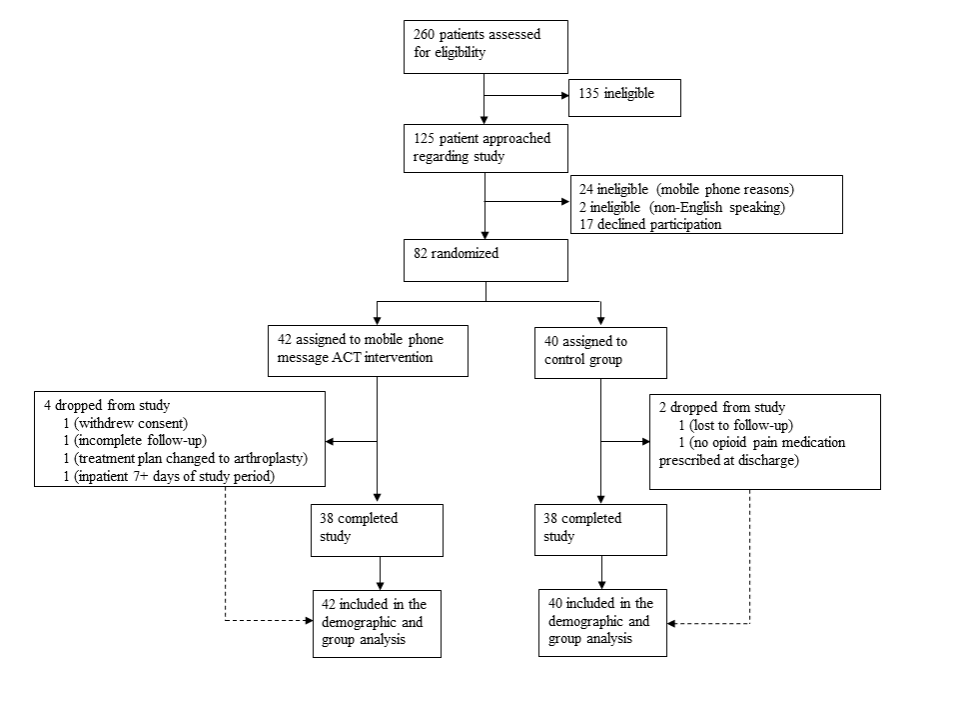
Consolidated Standards of Reporting Trials flowchart detailing the selection of eligible patients for study enrollment and their status through study completion. ACT: acceptance and commitment therapy.

A total of 125 individuals were approached regarding the study over the 5-month enrollment period between February 2019 and June 2019. Of the 125 individuals, 2 patients were excluded at this time, as they were non–English-speaking, and an additional 24 patients were excluded because they did not use mobile phone messaging or did not have a personal mobile phone. This resulted in a total of 99 eligible people who were presented the study, 17 of whom declined participation (Figure 1). Overall, 82 subjects were enrolled, and 6 dropped from the study after providing consent because of various issues: one patient lost to follow-up, one patient withdrew at follow-up, one patient had incomplete follow-up, one patient’s operative plan changed to arthroplasty, and 2 subjects remained inpatient for over 7 days of the study period (Figure 1). This resulted in a final population of 76 subjects (38 per study group). The enrollment period concluded once a powered sample for the primary aim was obtained. A breakdown of the subjects enrolled, reasons for excluding subjects, and subjects removed from the study after consent are presented in Figure 1. Participant demographics for the intervention and control groups are presented in Table 3. The analyses of all collected demographic factors showed no differences between the intervention and control groups in all factors such as subject age (*P*=.42), current psychiatric diagnosis (including depression, anxiety, bipolar type 1, obsessive-compulsive disorder, posttraumatic stress disorder, panic disorder, and attention-deficit disorder; *P*=.14), or substance abuse history (*P*=.10; Table 3). Furthermore, no differences between groups were found for injury type, disposition following discharge, method for reporting opioid medication consumption, preoperative opioid medication prescriptions, or preoperative PROs (Table 3).

### Opioid Pain Medication Use

No differences between groups were observed in the amount of opioid medication tablets or MME prescribed at discharge (tablets for the ACT group: mean 58.8, SD 27.3 vs tablets for the control group: mean 61.6, SD 22.0). A further breakdown of the medications prescribed to subjects within the study period is presented in Table 4. The mean opioid tablet use for subjects in the ACT-based intervention group was 26.1 (SD 21.4) tablets, whereas the control group used a mean of 41.1 (SD 22.0) tablets, resulting in 36.5% less tablets used by subjects receiving the ACT-based intervention (*P*=.004; Table 5). Similarly, subjects in the intervention group consumed a mean of 199.9 (SD 163.2) MME, on average, whereas the control group subjects consumed a mean of 307.0 (SD 166.0) MME, indicating 34.9% less MME used by subjects in the intervention group (*P*=.006; Table 5).

**Table 4:** Frequency of outpatient opioid pain medications prescribed by enrolled study group (N=82).

Medication	Morphine milliequivalents per tablet	Frequency	
		Acceptance and commitment therapy	Control
Hydrocodone-acetaminophen 5-325 mg	5	1	2
Hydrocodone-acetaminophen 10-325 mg	10	2	0
Hydromorphone 2 mg	8	6	6
Oxycodone 5 mg	7.5	27	27
Oxycodone-acetaminophen 5-325 mg	7.5	10	8
Total opioid prescriptions provided	N/A^a^	46	43

^a^N/A: not applicable.

**Table 5:** Opioid pain medication utilization by group during the 2-week study period.

Attribute	Opioid tablets dispensed			Opioid tablets consumed				Morphine milliequivalents consumed			
	ACT^a^ (n=38)	Control (n=38)	*P* value	ACT (n=38)	Control (n=38)	Decrease^b^ (%)	*P* value	ACT (n=38)	Control (n=38)	Decrease (%)	*P* value
Mean (SD)	58.8 (27.3)	61.6 (22.0)	.62	26.2 (21.4)	41.1 (22.0)	37	.004	199.9 (163.2)	307.0 (166.0)	35	.006
Median (minimum-maximum)	60.0 (10-146)	60.0 (15-120)	.62	21.0 (0-80)	43.5 (0-80)	37	.004	157.5 (0-600)	307.5 (0-600)	35	.006

^a^ACT: acceptance and commitment therapy.

^b^Calculated by the formula 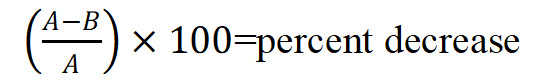.

### Patient-Reported Outcomes

PROMIS instrument *t*-score values for both ACT and control group subjects are presented in Table 6. At 2-week follow-up, the intervention group subjects reported lower postoperative PROMIS Pain Intensity 3A (mean 45.9, SD 7.2) and Pain Interference 8A (mean 56.6, SD 9.4) scores compared with the control group’s postoperative Pain Intensity 3A (mean 49.7, SD 8.8; *P*=.04) and Pain Interference 8A scores (mean 60.6, SD 8.2; *P*=.05; Table 6). No differences were observed between groups at 2-week follow-up in the PROMIS Pain Intensity 1A or PROMIS Emotional Distress-Anxiety 8A forms (Table 6).

**Table 6:** Mean Patient-Reported Outcome Measures Information System score and change within the 2-week study period by study group.

PROMIS^a^ instrument	Preoperative score					Postoperative score									Net score change										
	ACT^b^		Control		ACT				Control			*P* value		ACT					Control						*P* value
	Mean (SD)	Range	Mean (SD)	Range	Mean (SD)		Range	Mean (SD)		Range			Mean (SD)				Range	Mean (SD)				Range			
Pain Intensity 1A^c^	5.4 (2.9)	0 to 10	6.2 (2.6)	1 to 10	3.4 (2.2)		0 to 9	4.1 (2.4)		1 to 9	.22		−2.0 (2.9)			−10 to 7		−2.1 (2.3)		−9 to 2				.79	
Pain Intensity 3A	54.9 (7.3)	36.3 to 71.8	57.1 (8.2)	40.2 to 71.8	45.9 (7.2)		30.7 to 64.1	49.7 (8.8)		30.7 to 67.4	.04		−9.0 (8.5)			−25.5 to 10		−7.4 (7.7)			−23.9 to 6.1			.38	
Pain Interference 8A	63.6 (11.4)	40.7 to 77	66.1 (8.4)	40.7 to 77.0	56.6 (9.4)		40.7 to 72.0	60.6 (8.2)		40.7 to 77.0	.048		−7.1 (13.7)			−36.3 to 24.8		−5.4 (10.4)			−26.2 to 19.5			.55	
Emotional Distress-Anxiety 8A	56.5 (11.4)	37.1 to 80	56.5 (9.2)	37.1 to 76.7	51.5 (10.4)		37.1 to 75.4	52.3 (10.6)		37.1 to 76.7	.76		−4.9 (10.1)			−33.7 to 12.3		−4.2 (9.4)			−20.3 to 16.5			.74	

^a^PROMIS: Patient-Reported Outcome Measures Information System.

^b^ACT: acceptance and commitment therapy.

^c^Scores presented are raw numerical scores, as no *t-*score conversion is available for the selected instrument.

## Discussion

### Principal Findings

This randomized trial delivered an ACT-based intervention via an automated mobile messaging robot to postoperative orthopedic patients. The subjects who received the ACT-based mobile phone intervention used a lower number of opioid tablets and consumed less MME in the first 2 weeks after their injury. We also found that the intervention group reported less pain intensity and pain interference at the 2-week follow-up. These data demonstrate that ACT-based automated mobile messaging protocols may be effective in reducing the amount of opioid medication used and may positively affect postoperative PROs in patients undergoing operative fixation of their acute fractures.

### Effects on Opioid Use

Improved mood symptoms, less pain interference, and faster cessation of opioid pain medication are some of the recognized benefits of using ACT in clinic-based, interdisciplinary approaches to pain management after surgery [17,18]. Previous investigations have used automated mobile phone messaging robots to deliver PROs [20,23], improve communication with patients [27,31], deliver postoperative orthopedic care [32], and inquire about pain and opioid utilization [4,19,33]. In this study, we used ACT and a mobile phone messaging robot to assess whether these tools in combination could decrease opioid utilization and improve individuals’ perception of their early recovery from injury. Prior work has demonstrated a quicker time to opioid cessation and a decrease in postoperative opioid utilization (14% less in the ACT group) when used in office ACT-based treatments [17,18]. Subjects receiving the ACT intervention via automated mobile phone messages reported over 36% less opioid tablets and more than 34% less MME consumed than corresponding control subjects who did not receive ACT. Our findings suggest that software-based communication using ACT through a mobile phone has the potential to have a large impact on the utilization of postoperative pain medication by patients in the first weeks after surgery for fractures. Further study is required to determine if these effects are long lasting and to determine which injuries and patients receive the greatest benefit. In addition, future investigations and trials should consider the effect of software delivery of ACT and other behavioral therapies on different cohorts of patients.

### Effects on PROs

PROs, such as PROMIS, allow patients to quantify aspects of their orthopedic condition in a standardized fashion [6,7]. These are important tools for determining the efficacy of health care treatments and assessment of clinical research and can be used in determining compensation for health care services provided [6-8]. The National Institutes of Health developed the PROMIS tools to advance PROs by creating question banks that could be used for many major health issues [9]. We found that despite less utilization of opioids, subjects in the ACT-based intervention group reported less pain intensity and pain interference at 2 weeks. This most likely does not represent a clinically important difference based on the SD methodology used in prior works with PROMIS tools (Table 6), but it at least suggests that the intervention group did not experience greater pain [34]. There were no other differences between study groups in the other domains at the 2-week follow-up. Previous studies have reported that patients who consume more opioid medication report higher pain at both short- and long-term time points, which is reflected in our findings for both PROMIS pain intensity 3A and the employed pain interference measure [35,36]. Future research efforts may benefit from employing alternative PRO measures to identify the effects of ACT-based interventions, including assessment of psychologic flexibility. Future research may also consider possible modifications of our study protocol to include a longer intervention period and more than one follow-up data point. Future work may also consider designing an ACT-based tool that is more focused on demonstrating an effect on PROs.

### Limitations

Several limitations were present in this study. First, we were limited to a single level 1 trauma center, which may affect the reproducibility of our results across other health care settings. Next, the exclusion criteria for this study were extensive, and thus, the results may not be generalizable to the entire scope of orthopedic trauma patients. We attempted to include a diverse set of injuries and yet excluded patients with a high likelihood of confounding problems from open fractures or prolonged initial hospitalization. Future studies assessing the effects of ACT-based interventions similar to ours should aim for less restrictive exclusion criteria to apply this intervention to a larger, more diverse population. The research assistants were not blinded to the patients’ study group. In addition, patients understood the outcomes of interest in this study, which could be susceptible to reporting bias. In addition, participants were not blinded to their treatment group. The lack of blinding could potentially introduce response or reporting bias, making this a potential area of improvement for studies seeking to follow the present methodology. This could be accomplished through the implementation of a control messaging protocol. Moreover, a retrospective chart review was used to obtain several patient factors, including comorbid conditions and dispensing of preoperative outpatient opioid medication prescriptions. The collection of information in this manner relies on accurate charting and transfer of documents from outside institutions, which may have been incomplete.

### Conclusions

In this study, delivering an ACT-based intervention via an automated mobile messaging robot in the acute postoperative period decreased opioid utilization in orthopedic trauma patients in the first 2 weeks after their injury. Subjects in the ACT-based intervention group also reported lower pain intensity and pain interference after 2 weeks, although this likely did not represent a clinically important difference. Future studies may apply this intervention in other patient populations to assess its efficacy on a larger scale and may include assessment of pain and opioid use in a longer time frame after injury.

## Appendix

Multimedia Appendix 1 CONSORT-EHEALTH checklist (V 1.6.1).

Multimedia Appendix 2 Patient-Reported Outcome Measure Information System tools completed by the study subjects.

Multimedia Appendix 3 Acceptance and commitment therapy–based automated mobile phone messaging protocol.

## Abbreviations

ACT: acceptance and commitment therapy
MME: morphine milliequivalents
POD: postoperative day
PRO: patient-reported outcome
PROMIS: Patient-Reported Outcome Measure Information System
