# Supplementary material for: Acceptance and Commitment Therapy Delivered via a Mobile Phone Messaging Robot to Decrease Postoperative Opioid Use in Patients With Orthopedic Trauma: Randomized Controlled Trial
Source: J Med Internet Res. 2020 Jul 29;22(7):e17750. doi: 10.2196/17750 (PMC7458063; doi:10.2196/17750)
Supplement: Multimedia Appendix 2 [file jmir_v22i7e17750_app2.pdf]

Pain Intensity – 1a

Please respond to the question by marking one box.

Pain Intensity
In the past 7 days…

		0	1	2	3	4	5	6	7	8	9	10
Global07	How would you rate your pain on average?...	☐ **No pain**	☐	☐	☐	☐	☐	☐	☐	☐	☐	☐ **Worst imaginable pain**

© 2008-2017 PROMIS Health Organization (PHO)

Pain Intensity – Scale

Please respond to each item by marking one box per row.

	**In the past 7 days…**	**Had no pain**	**Mild**	**Moderate**	**Severe**	**Very severe**
PAINQU6	How intense was your pain at its worst?....	☐ 1	☐ 2	☐ 3	☐ 4	☐ 5
PAINQU8	How intense was your average pain?.........	☐ 1	☐ 2	☐ 3	☐ 4	☐ 5
		**No pain**	**Mild**	**Moderate**	**Severe**	**Very severe**
PAINQU21	What is your level of pain right now?........	☐ 1	☐ 2	☐ 3	☐ 4	☐ 5

© 2008-2017 PROMIS Health Organization and PROMIS Cooperative Group

Pain Interference – Short Form 8a

Please respond to each question or statement by marking one box per row.

In the past 7 days…

		Not at all	A little bit	Somewhat	Quite a bit	Very much
PAININ9	How much did pain interfere with your day to day activities?	☐ 1	☐ 2	☐ 3	☐ 4	☐ 5
PAININ22	How much did pain interfere with work around the home?	☐ 1	☐ 2	☐ 3	☐ 4	☐ 5
PAININ31	How much did pain interfere with your ability to participate in social activities?	☐ 1	☐ 2	☐ 3	☐ 4	☐ 5
PAININ34	How much did pain interfere with your household chores?	☐ 1	☐ 2	☐ 3	☐ 4	☐ 5
PAININ12	How much did pain interfere with the things you usually do for fun?	☐ 1	☐ 2	☐ 3	☐ 4	☐ 5
PAININ36	How much did pain interfere with your enjoyment of social activities?	☐ 1	☐ 2	☐ 3	☐ 4	☐ 5
PAININ3	How much did pain interfere with your enjoyment of life?	☐ 1	☐ 2	☐ 3	☐ 4	☐ 5
PAININ13	How much did pain interfere with your family life?	☐ 1	☐ 2	☐ 3	☐ 4	☐ 5

© 2008-2018 PROMIS Health Organization (PHO)

Emotional Distress – Anxiety – Short Form 8a

Please respond to each question or statement by marking one box per row.

In the past 7 days…

		Never	Rarely	Sometimes	Often	Always
EDANX01	I felt fearful	☐ 1	☐ 2	☐ 3	☐ 4	☐ 5
EDANX40	I found it hard to focus on anything other than my anxiety	☐ 1	☐ 2	☐ 3	☐ 4	☐ 5
EDANX41	My worries overwhelmed me	☐ 1	☐ 2	☐ 3	☐ 4	☐ 5
EDANX53	I felt uneasy	☐ 1	☐ 2	☐ 3	☐ 4	☐ 5
EDANX46	I felt nervous	☐ 1	☐ 2	☐ 3	☐ 4	☐ 5
EDANX07	I felt like I needed help for my anxiety	☐ 1	☐ 2	☐ 3	☐ 4	☐ 5
EDANX05	I felt anxious	☐ 1	☐ 2	☐ 3	☐ 4	☐ 5
EDANX54	I felt tense	☐ 1	☐ 2	☐ 3	☐ 4	☐ 5

© 2008-2016 PROMIS Health Organization and PROMIS Cooperative Group
