# Supplementary material for: Acceptance and Commitment Therapy Delivered via a Mobile Phone Messaging Robot to Decrease Postoperative Opioid Use in Patients With Orthopedic Trauma: Randomized Controlled Trial
Source: J Med Internet Res. 2020 Jul 29;22(7):e17750. doi: 10.2196/17750 (PMC7458063; doi:10.2196/17750)
Supplement: Multimedia Appendix 3 [file jmir_v22i7e17750_app3.pdf]

Acceptance and Commitment Therapy based automated mobile phone messaging protocol

Postoperative Day	ACT Core Principle Utilized	Morning Message	Evening Message
1	**Values:** Know what matters most	Maintaining focus on what you value most in life is sometimes difficult after surgery. Do not let the momentary discomforts due to surgery take away from what you want most in life. Pick 3 things that matter most to you in life. Remind yourself of these 3 things you value most during your recovery process.	Stop for a moment and remember the 3 values you identified earlier today. Remind yourself how important these values are in your life. As your day comes to an end, remember that YOU are in control of the thoughts that exist in your mind. We encourage you to spend time thinking about your 3 core values identified earlier today.
2	**Acceptance:** Setting expectation that pain is a part of surgery	Pain is a part of recovering from surgery. Pain is something you can choose to observe and not something that requires you to act. Your life goals, values, and subsequent actions determine what your life looks like after surgery.	Feelings of pain and feelings about your experience of pain are normal after surgery. Acknowledge and accept these feelings as part of the recovery process. Remember how you feel now is temporary and your healing process will continue. Call to mind pleasant feelings or thoughts that you experienced today.
3	**Present Moment Awareness:** Mindfulness and awareness for our thoughts in the present moment	Awareness of the present moment and your breathing may change with pain-related emotions or thoughts. Remember you can always count on your breathing to bring you back to the present moment and help you move through your current experience of pain. Try utilizing this breathing app to help: examplelink.com	Breathing helps bring awareness about how you are reacting to thoughts in the moment. You can also use breathing to help you focus on what you want in life and what is important to you. End your day in a calm state. This breathing app can help you get there. examplelink.com
4	**Values:** Know what matters most	Pause and reflect on the aspects of your life that bring meaning and purpose. Think of 3 life goals or motivations that you want to focus on moving forward. The journey to recovery can be difficult but use what gives your life meaning as a guide for the actions you take during recovery.	Before your day ends think about how the values you have identified in life were part of the things you did today. Think about how you can do things differently tomorrow so that you can build upon what you accomplished today.

5	**Self-as-**Context: Awareness of what is being observed and noticed by ourselves	We cannot change that a feeling or thought may arise, but we can choose how we respond to our feelings and thoughts. Remember that dwelling on pain, discouraging feelings, and thoughts after surgery are NOT consistent with your life goals and values. Observe things that try to move you away from your values and only act on things that are compatible with who you want to be and what matters to you.	Acknowledge your thoughts and feelings today about how your recovery is progressing. Remember that your surgery and your recovery do not define you. Your life is much larger and fuller than just your recovery process. Spend some time tonight thinking about something or someone important in your life.
6	**Committed Action:** Doing what it takes to live according to our values	Healing after surgery requires you to act. We previously discussed your life goals, meaning, and purpose. Take action today and move closer towards what you want in life. Recognize that pain may be present but make the choice that it will not impede your progress toward what you really want in life. Be present in the moment and ensure your actions remain true to what you want most. All actions you make no matter how small, are an important stepping stone on your road to recovery.	Now that your day is winding down, reflect on what motivated your actions throughout the day. Intrusive thoughts, emotional distress, and pain may show up as you are recovering, but these thoughts and feelings do not need direct the actions you take. Remember that you get to decide how to live in a way that reflects what matters to you.
7	**Acceptance:** Setting expectation that pain is a part of surgery	Recovery is a process and pain is a part of this process. By accepting this you can control your actions related to pain and choose how big of a role pain plays in your recovery. Limiting your reaction to pain by taking less pain medication and staying on track with your recovery activities are examples how you can succeed with this.	Reflect back on today. Think about the role that pain played in the activities you took part in. Take notice of any instances where pain slowed down your recovery and try to improve on those tomorrow. Remember, pain is part of the recovery process, but not what dictates it. Continue to move forward with a focus on what matters most to you in life and make the pain take a back seat. Find one thing you can be thankful for today.
8	**Defusion:** Watch your thinking and interact with thoughts in a way consistent with your values.	If you ever feel pain after surgery know that the feeling is real but what it actually represents is not what you might think. Our mind is capable of making us feel pain, even though there is no damage going on in our body. Pause, become more aware in the moment and chose a skillful response that will help you move toward your overall goals and values.	Pain, discomfort and anxiety are common after surgery. Remember you do not need to avoid these feelings and thoughts. Recognize they will be a part of your recovery and move past them as they will only be momentary. Take a deep breath, and as you breathe out allow yourself to focus on something you are thankful for today.

9	**Values:** Know what matters most	Remaining actively engaged in your recovery after surgery can be difficult. In these moments it is important to focus on the things that matter most to you in life. Take time to reflect on your goals and values and allow them to support you in the moments you find it difficult to remain positive during the recovery process.	Take several minutes this evening and remind yourself of what matters most to you in life. Imagine things you can do tomorrow to move closer to these values. Remember to define your recovery by how you move towards what matters most to you in life and not by the momentary feelings of discomfort that everyone has after surgery.
10	**Acceptance:** Setting expectation that pain is a part of surgery	Recovery following surgery is imperfect. Emotional distress and pain often play a role, but accepting their presence helps diminish their possible negative impact on your recovery. What you value most in life and your subsequent actions are what truly pave the road to recovery. Staying consistent with your goals and values is your key to success.	You may be feeling pain and discomfort during your recovery. Remember this is part of the recovery process for everyone. Willingness to notice this discomfort and accepting that this is just part of the process, rather than wasting energy and strength trying to avoid it, keeps you moving toward what matters most to you in life
11	**Committed Action:** Doing what it takes to live according to our values	Engaging in activities that we enjoy can be difficult due to pain and emotional distress after surgery. Anything that brings you joy, and meaning is important. Whether you enjoy a specific TV show, walking or just being outside, dinner with friends, or reading a good book, do something today that you enjoy.	Take some time before your day ends and do 1 thing that is just for you. This could be time with someone important to you, enjoying the outdoors, or watching your favorite TV show. Despite how your body may be feeling, determine one thing you want to do before your day ends and take time to enjoy life.
12	**Self-as**-Context: Awareness of what is being observed and noticed by ourselves	Remember who you are today. Remember your values, goals and aspirations in life. Notice that the thoughts, emotions, and pain you may feel after surgery can blur your sense of self and keep you from doing things consistent with your values. Even though these things may show up, they don't need to determine what you do. Not reacting to these thoughts or feelings about yourself or your pain is success when recovering from surgery.	Acknowledge what you think and feel today regarding how you feel after surgery. Remember that thoughts and feelings about your pain may show up, but they don't have to keep you stuck. Instead, allow yourself to focus on thoughts that center around who you are and who you want to be.
13	**Committed Action:** Doing what it takes to live according to our values	Taking actions, moment by moment, that move you toward the things that matter most to you in life is the most effective way to move through your recovery process. Carrying out any action that is in-line with your goals and values is a major win, just like adding a correct new piece to a puzzle.	As your day comes to an end, think back on the wins you had today. Use these wins to motivate you and keep you moving when things are difficult. The wins will keep coming and the puzzle will continue to take shape as long as you keep taking actions that reflect the things that matter most to you in life.

14	**Committed Action:** Doing what it takes to live according to our values	Happiness and having fun are values that are sometimes easy to forget during recovery from surgery. Reflect on what activities you can do today that bring joy and meaning to your life. Make it your goal today, and every day during recovery, to do at least one thing that brings your life joy and meaning.	With your day in mind, remember that activities that bring you joy and represent your values are important to your recovery. Taking part in these activities is as powerful as anything you can do to help you make progress during your recovery.

ACT: Acceptance and Commitment Therapy
